# A low dose of curcumin-PDA nanoparticles improves viability and proliferation in endoneurial fibroblasts and Schwann cell cultures

**DOI:** 10.1186/s11671-024-04023-7

**Published:** 2024-05-07

**Authors:** Lucia Vázquez Alberdi, Marcela Martínez-Busi, Eloisa Arrarte, Carolina Echeverry, Miguel Calero, Alejandra Kun

**Affiliations:** 1 Laboratorio de Biología Celular del Sistema Nervioso Periférico, Departamento de Proteínas y Ácidos Nucleicos, Instituto de Investigaciones Biológicas Clemente Estable, Montevideo, Uruguay; 2 Laboratorio de Acústica, Facultad de Ciencias, Instituto de Física, UdelaR, Montevideo, Uruguay; 3 Plataforma Química Analítica, Instituto de Investigaciones Biológicas Clemente Estable, Montevideo, Uruguay; 4 Área Fisicoquímica, Facultad de Química, UdelaR, Montevideo, Uruguay; 5 Departamento de Neurobiología y Neuropatología, Instituto de Investigaciones Biológicas Clemente Estable, Montevideo, Uruguay; 6 Unidad de Encefalopatías Espongiformes, UFIEC, CIBERNED, Instituto de Salud Carlos III, Madrid, Spain; 7 Sección Bioquímica, Facultad de Ciencias, Universidad de La República, Montevideo, Uruguay

**Keywords:** Curcumin, Polydopamine-nanoparticles, Hormesis, Viability, Proliferation

## Abstract

Curcumin is a polyphenol extracted from *Curcuma longa*’s roots. Low doses of curcumin are related to anti-inflammatory, antioxidant, and neuroprotective effects, while high doses are used for their lethality. This diversity of behaviors allows us to understand curcumin as a compound with hormetic action. Due to its strongly hydrophobic character, curcumin is often solubilized in organic compounds. In this way, we have recently reported the undesirable and occasionally irreversible effects of alcohol and DMSO on the viability of primary Schwann cell cultures. In this scenario, the use of nanoparticles as delivery systems has become a successful alternative strategy for these compounds. In the present work, we describe the structure of Polydopamine (PDA) nanoparticles, loaded with a low dose of curcumin (Curc-PDA) without the use of additional organic solvents. We analyzed the curcumin released, and we found two different forms of curcumin. Small increased cell viability and proliferation were observed in endoneurial fibroblast and Schwann cell primary cultures when Curc-PDA was steadily supplied for 5 days. The increased bioavailability of this natural compound and the impact on cells in culture not only confirm the properties of curcumin at very low doses but also provide a glimpse of a possible therapeutic alternative for PNS conditions in which SCs are involved.

**Supplementary Information:**

The online version contains supplementary material available at 10.1186/s11671-024-04023-7.

## Introduction

Curcumin is a polyphenol extracted from the roots of the *Curcuma longa* plant, native to India [1–4]. It is a yellow-orange solid with diverse applications, with a broad spectrum of action depending on its concentration and the administration time [5–12]. It has been reported that low doses of curcumin are related to anti-inflammatory [13, 14], antioxidant [9, 15–17], and neuroprotective effects [10, 11, 18, 19]; whereas, at high concentrations, it has lethal effects, is used as a potent anti-tumor agent [5–8, 20, 21]. This diversity of behaviors allows us to understand curcumin as a hormetic compound. Thus, at low doses, it has cellular effects that promote cell development, while at high doses it promotes cell death [22–24].

Due to its hydrophobic character, curcumin needs to be solubilized in organic solvents, the most conventionally used being ethanol and dimethyl sulfoxide (DMSO) [20, 21, 25–28]. Unfortunately, these vehicles present effects by themselves (especially visible at prolonged exposure times), more or less detectable depending on the biological models used [29–32]. Recently, we have reported effects on the viability and proliferation of primary Schwann cell cultures of both ethanol and DMSO, irreversible in a pathological context, after 6 days of treatment [30].

Given the great versatility of curcumin, the limitations of its conventional vehicles, and its low bioavailability, several strategies have emerged to improve its delivery. The use of cyclodextrin/cellulose nanocrystals coated with curcumin [33, 34], curcumin in polyethylene glycol [35, 36], nanosuspension in tween-80 [37], in chitosan/aloe film [38], conjugated to polyacetal [39], constitute some different strategies reported to the vehicle this compound, although there are many others [40–42].

Among other alternative approaches, the use of nanoparticles as delivery systems is a successful strategy [43–45]. Recently, polydopamine (PDA) nanoparticles have been used not only as coatings and surface functionalization [46, 47] but also as vehicles of different compounds [48–52]. Some studies have used PDA to vehicle curcumin with some differences. Pan et al. 2020 created carrier-free curcumin nanoparticles of different concentrations between 4 and 50 μg/ml (approximately between 11 μM and 136 μM), which they subsequently coated with PDA, demonstrating that these curcumin-loaded nanoparticles are stable structures, with curcumin release dependent on pH variations [49]. In 2021, Su et al. produced PDA nanoparticles, and then exposed them to curcumin (around 1.13 mM curcumin), demonstrating their antioxidant and antibacterial properties in yeast cultures [50]. Zhao et al., 2022 use PDA nanoparticles coating curcumin loaded with poly L-lactic acid for chemo-photo thermal therapy of osteosarcoma. The work demonstrates that by thermo-activation of nanoparticles loaded with approximately 1 mg/ml curcumin (⋍ 2.72 mM), their release in human osteosarcoma cultures (MG-63) is possible, depending on the pH of the intracellular medium [51]. Recently, Lei et al., 2023 coated a rabies virus glycoprotein (RVG29 peptide) to PDA nanoparticles with 0.3 mmol curcumin previously dissolved in PEG and DMSO (approximately 2.24 mM), to target the nanoparticles to the murine brain. The work explores the antiaggregatory effects of curcumin on α-synuclein in different experimental models (Balb/c mice, *C. elegans*, and PC12 cell culture). The results further demonstrate a decrease in oxidative stress levels and apoptosis upon delivery of curcumin through these nanoparticles [52]. The literature thus points to the sensitivity and dependence of curcumin release from PDA nanoparticles in response to the pH of the medium. The different effects of curcumin on the dose used, evidence its hormetic action, especially when considering the use of these nanoparticles at the biological level [50–52].

Primary culture approaches can be used to understand metabolic responses during specific stimuli or insults. In the peripheral nerve fiber, in addition to neurons, Schwann cells (SC) and endoneurial fibroblasts (EFB) are present. Establishing primary cultures of SCs and EFBs allows us to understand the metabolic responses of in vitro proliferation. In the healthy nerve fiber, SCs and EFBs show a very low proliferative rate, mainly activating it in response to damage [53, 54]. However, under neurodegenerative conditions, proliferation is a frequent event in response to punctate or permanent injury [16, 33].

In the present work, we propose a new fabrication and loading protocol for Curc-PDA, without using organic solvent to load curcumin into PDA. This protocol incorporates a key dialysis step to eliminate possible pH variations outside the physiological range. We describe the structure of PDA nanoparticles, loaded or not with curcumin, by Transmission/Scanning Electron Microscopy (TEM and SEM) and Diffraction Light Scattering (DLS). We analyze their loading and unloading dynamics with curcumin, characterizing the released compounds by Ultra-High-Pressure-Liquid Chromatography with a Mass-Spectrometry (UHPLC-MS). Finally, we tested the safety of PDA as a vehicle (without curcumin) and the functional dynamics of nanoparticles loaded with low doses of curcumin in endoneurial fibroblast and Schwann cell cultures, evaluating their impact on cell viability and proliferation for prolonged periods of time.

## Materials and methods

### PDA fabrication and curcumin loading

**Fig. 1:**
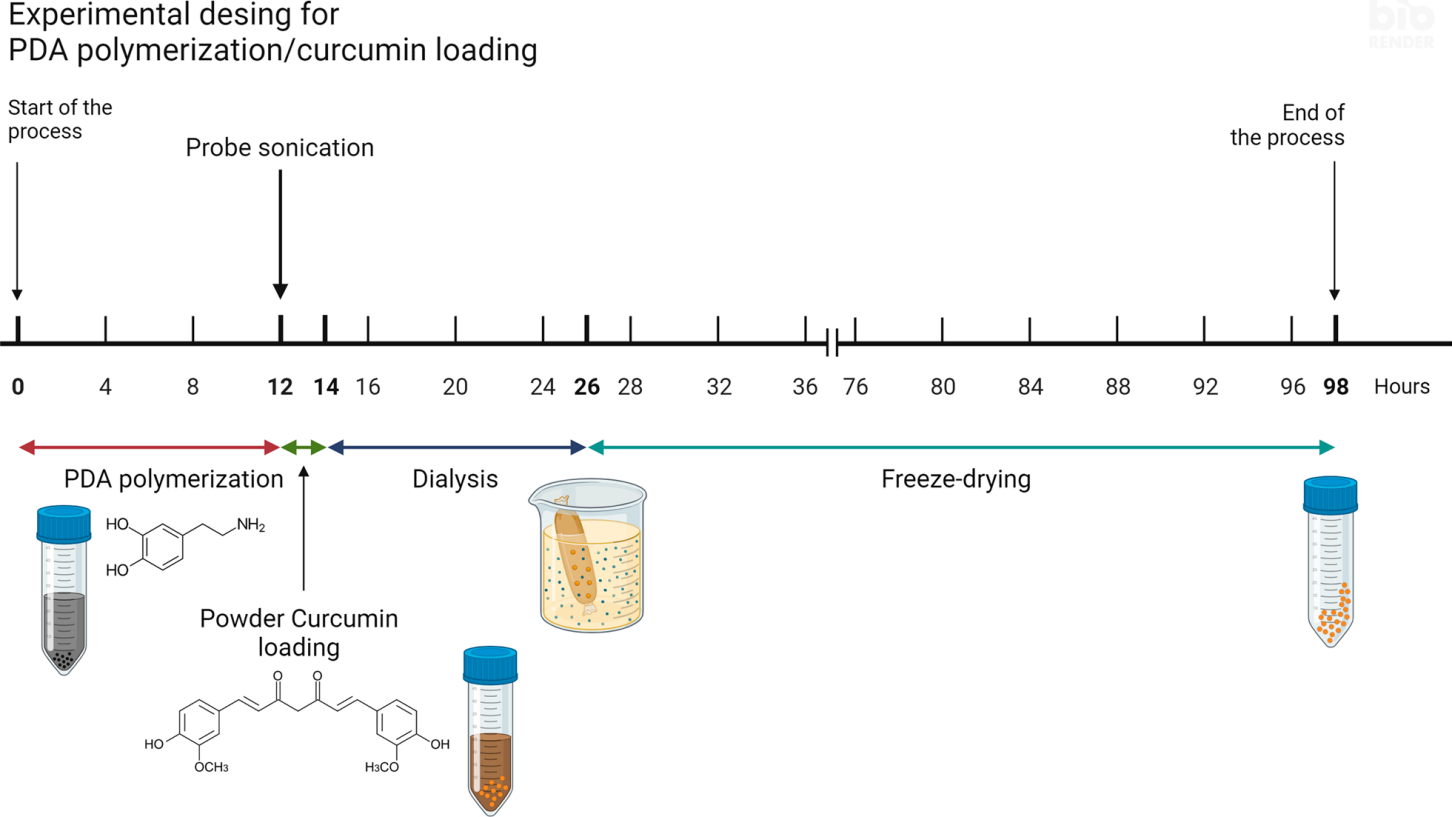
Experimental design of PDA polymerization and curcumin loading. Polydopamine (PDA) nanoparticles were polymerized in TRIS–HCl buffer pH = 8.5, 10 mM, for 12 h at RT, protected from light and under constant agitation. Then, a stem sonication was performed, followed by loading with curcumin powder (without organic solvents) for 2 h at RT, protected from light and under constant stirring. Dialysis was performed for 12 h at RT, protected from light and under constant stirring in a 14 kDa membrane. At the end of the time, the PDA or Curc-PDA was lyophilized for 72 h. The resulting powder is stored at 4 °C protected from light until use. The scheme was created with BioRender.com

Polydopamine nanoparticles (PDA) were polymerized with 10 mM dopamine hydrochloride (Cat#: H8502, Sigma-Aldrich, Taufkirchen, Germany) in TRIS–HCl (Cat#: 1185-53-1, Sigma-Aldrich, Taufkirchen, Germany) buffer pH = 8.5, 10 mM, for 12 h at room temperature (RT), protected from light and under constant agitation. Then, a probe sonication was performed, followed by loading with curcumin for 2 h at RT, protected from light and under constant stirring. For this, we incorporated the curcumin powder directly into the PDA solution so the curcumin is finally at a concentration of 10 mM. Dialysis was performed for 12 h at RT, protected from light and under stirring in a 14 kDa membrane (Cat#: D9527, Sigma-Aldrich, Taufkirchen, Germany). Finally, the PDA or Curcumin in PDA (Curc-PDA) was lyophilized for 72 h. The resulting powder was stored at 4 °C protected from light until use. The schematic model is shown in Fig. 1.

### PDA and Curc-PDA transmission and scanning electron microscopy

The morphology and size of PDA and Curc-PDA in an aqueous solution were determined by transmission electron microscopy (TEM). For unloaded PDA, a suspension of 1.2 mg/ml (10 mM) was made in distilled water, which was then diluted 100-fold (100 μM). For Curc-PDA, the same suspension was made, assuming a similar concentration, of the loaded nanoparticles. A sample drop (10 μl) of PDA or Curc-PDA in distilled water was deposited on a 300-mesh carbon-coated copper mesh, dried for 20 min at RT, and then, the sample was observed by TEM. The surface morphology of PDA and Curc-PDA were also observed by scanning electron microscopy (SEM). Using the same solutions as for TEM, 50 μl was placed in blocks on a double-sided tape, dried at RT, and then metalized for SEB visualization.

### Diffraction light scattering

The hydrodynamic diameter of the synthesized nanoparticles was determined using Dynamic Light Scattering (DLS) with a Nanoptic 90 instrument (BetterSize, China) operating at a wavelength (λ) of 635 nm and a scattering angle (θ) of 90°. The measurements were conducted at 25 °C, with the nanoparticles dispersed in a culture medium. Before analysis, the samples were sonicated in three cycles of 30 s each to ensure proper dispersion. The mean particle size was determined by averaging three measurements from a single sample.

### HPLC experiments

#### UHPLC-MS analyses

LC–MS analyses were performed by an Ultimate 3000 UHPLC instrument coupled with an ISQ EC mass spectrometer, equipped with an electrospray ion source and a single-quadrupole analyzer (Thermo Fisher Scientific, Cambridge, MA, USA). A C18, Luna® Phenomenex, 5 µm 100 Å (150 × 4.6 mm) (Phenomenex, Torrence, CA, USA) was used for chromatographic separation. The mobile phase was composed of solvents A (Water, 0.1% HCOOH) and B (Acetonitrile, 0.1% HCOOH). An isocratic mode (30:70) was used. The flow rate was set at 0.2 mL/min and the column was maintained at 30 °C for the entire run. Fifty microliters were injected. Analyses were performed using the positive ionization mode selecting the following m/z: 91, 137, 154, 369. The collision energy (CE) was 20 eV. Mass spectrometry parameters were: spray voltage (V) 3000, sheet gas (arb) 28.8 psig, aux gas (arb) 3.2 psig, ion transfer tube temperature 300 °C, and vaporizer temp 117 °C. Peak areas were measured by using the Chromeleon software (Thermo Fisher Scientific).

#### Curcumin quantification in PDA

To determine the load of curcumin on PDA nanoparticles, 1 mg/ml solutions of Curcumin alone, unloaded PDA, and Curc-PDA in methanol were created. We then searched for curcumin by UHPLC-MS at m/z = 368 ion. We made three independent experiments, with duplicates of each sample. To calculate the curcumin concentration within the PDA; we used the area under the curve of the standard curcumin peak, of known concentration, and obtained the relationship to the area under the curve of the curcumin peak in the Curc-PDA sample.

#### Release-retention dynamics

To evaluate how curcumin was released from PDA to the culture medium, we studied its release dynamics during 24 h. To do so, we generated a 1 mg/ml solution of Curc-PDA in a culture medium and separated the solution into different tubes to have the samples at different times: 0.5, 1.0, 1.5, 2.0, and 24 h. At each time, the tube was centrifuged, and a sample of the supernatant was taken to look for curcumin released into the culture medium. Then, the medium was removed, replaced by an equal volume of methanol, and centrifuged one more time. As a result, we obtained the curcumin retained in the PDA, which was removed from the nanoparticle by methanol. Both samples were analyzed by UHPLC-MS at m/z = 368 ion, in three independent experiments.

### Animals

C57BL wild-type (Wt) mice were obtained from Jackson Laboratories (JAX stock #002504, Jackson Laboratories, Bar Harbor, ME, USA). The colony was maintained at the Clemente Estable Biological Research Institute (IIBCE, MEC) bioterium. The local ethics committee approved all the experiments and procedures (Comisión de Ética en el Uso de Animales (CEUA), IIBCE, Uruguay, protocol number: 002a/10/2020). The regulations and guidelines were followed according to Uruguayan Law number 18611 (https://www.impo.com.uy/bases/leyes/18611-2009/8). Mice were housed in a controlled environment (12 h light/dark cycle) and a mean temperature of 21 ± 3 °C with food and water-free access. This work used postnatal male mice 5 days old (n = 5 for each group).

### Endoneurial fibroblasts and Schwann cell primary culture

Sciatic nerve fibers were dissected as previously described [30]. Briefly, after decapitation, both sciatic nerves were dissected using surgical scissors. The nerves were then immersed in Dulbecco’s Modified Eagle’s Medium (Cat#: DMEM-HSPTA, Capricorn, Ebsdorfergrund, Germany) supplemented with 10% Bovine Serum (FBS, Cat#: 26140079, Gibco™, Waltham, MA, USA); 5 µg/mL penicillin, 5 µg/mL streptomycin, 10 µg/mL neomycin (PSN 1X, Cat#: 15640055, Gibco™, Waltham, MA, USA). Immediately, the epineurium was removed and the fibers were teased under a stereoscopic microscope.

After, the fibers were incubated for 30 min at 37 °C to collagenase (WD: 225 µg/mL, Cat#: C9407, Sigma-Aldrich, Taufkirchen, Germany) in DMEM supplemented, and 5 mM CaCl2. The fibers were centrifugated, the supernatant was removed and it was incubated for 30 min at 37 °C to trypsin (WD: 0.25%, Cat#: 15,090,046, Gibco™, Waltham, MA, USA) in DMEM with PSN 1X. Then, it was centrifugated and finally, the pellet was resuspended in DMEM supplemented, plated, and cultured at 37 °C and 5% CO_2_. The next days, the culture was evaluated by an inverted light microscope for Schwann cells (SC) and endoneurial fibroblast (EFB) growth.

The medium was replaced every 48 h, and after one week to obtain SC and EFB enrichment cultures, we performed the cold jet procedure [55]. For SC culture, the medium was supplemented with 8 µM forskolin (Cat#: F6886, Sigma-Aldrich, Taufkirchen, Germany) and 20 µg/mL bovine pituitary extract (Cat#: P1167, Sigma-Aldrich, Taufkirchen, Germany). The last one was used in the experiments and was not further than passage 3.

### Determination of the concentration of PDA suitable for cultures

To determine the concentration without affecting the cultures, viability, and proliferation assays were performed. The viability of the cultures was studied by 3-(4,5-Dimethylthiazol-2-yl)-2,5 Diphenyltetrazolium Bromide (MTT) assay and the proliferation was carried out with CyQUANT™ Cell Proliferation Assay (Cat#: C7026, Invitrogen, Eugene, OR, USA). For both assays, 1 × 10^5^ cells per well were seeded, in a 96-well plate. 24 h later, the medium was removed and replaced by a medium containing different concentrations of PDA (0.32; 0.64; 1.93; 9.63; 19.25; 192.50; 1925 mg/l). The concentrations were evaluated in triplicate and three independent experiments were carried out. The treatment was conducted for five days, with medium changes every 24 h. We used untreated control as a control, being 100% of viability.

Every day, for viability the medium was removed and replaced by culture medium with the reagent MTT (Cat#: M6494, Invitrogen, Eugene, OR, USA), work dilution (WD): 0.5 mg/mL. This pale yellow, water-soluble compound is reduced in the presence of living cells, precipitating as formazan (violet-blue crystals, insoluble in water) [56]. The cells were incubated for 2 h at 37 °C and 5% CO_2_. Then, the medium was removed and the cells were lysed with DMSO to release and solubilize the formazan crystals followed by absorbance measurements at 570 nm and 650 nm (background) in the Varioskan® (Varioskan® Flash, Thermo Fisher Scientific, Waltham, MA, USA). For proliferation, each day the medium was removed and replaced by 200 μl of the mixing kit per well, and after 5 min, we performed fluorescence measurements exiting at 480 nm and measuring the emission at 520 nm, in the Varioskan®.

### Treatment with Curc-PDA

To study the effect of curcumin on culture viability and proliferation, a concentration of 0.05 μM curcumin in PDA was used, below the PDA concentration limit previously determined. Using the same approach as in the previous section, the analysis of MTT and CyQUANT was performed every 24 h, following the effect on the cultures for 5 days. We used PDA without curcumin as a control of 100% viability and made three independent experiments.

### Statistical analysis

The normality of the data obtained was evaluated by the Shapiro–Wilk test. The analysis of released and retained compounds was performed with paired student t-test or Wilcoxon test, comparing the values obtained for each time analyzed. Within each day, Viability and proliferation values for the different PDA concentrations were evaluated with one-way ANOVA, Bonferroni post hoc test, or Kruskal Wallis test, with Dunn’s correction. Viability and proliferation values of cultures treated with PDA or with Curc-PDA were evaluated per day, with the unpaired Student’s t-test or with the Mann–Whitney test. All tests were applied using a two-tailed distribution and the results were considered significant at an alpha level of 0.05. Statistical analysis was performed with GraphPad Prism version 8.0.0 (RRID: SCR_002798, GraphPad Software, San Diego, CA, USA).

## Results

### PDA structure and quantification of curcumin loading

**Fig. 2:**
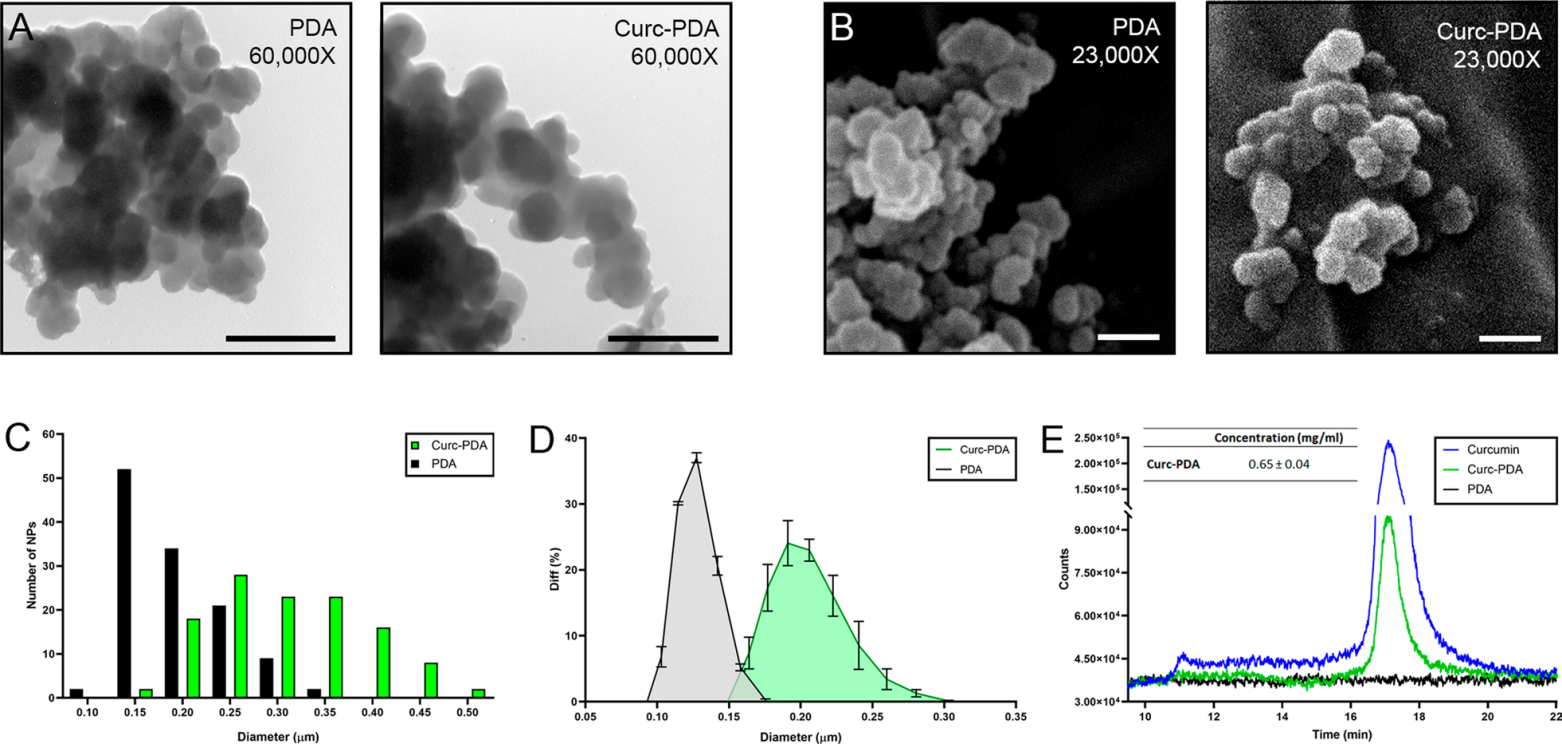
PDA structure and quantification of curcumin loading. **A** Transmission electron microscopy images of 100 µM unloaded (PDA) and curcumin-loaded (Curc-PDA) Nanoparticles. **B** Scanning electron microscopy images of 100 µM PDA and Curc-PDA. **C** Frequency diagram of the diameters obtained by electron microscopy. **D** PDA and Curc-PDA diameters obtained by diffraction light scattering analysis. **E** Spectra obtained by UHPLC-MS of samples solubilized in methanol at 1 mg/ml of curcumin, PDA, or Curc-PDA. By area integration, it is obtained that the concentration of curcumin loaded in the nanoparticles is expressed as mean ± SEM. Scale: 0.5 µm

The structure of unloaded and curcumin-loaded Polydopamine nanoparticles was determined by TEM and SEM (Fig. 2A and B). From the images obtained, the diameters of the nanoparticles were calculated. The unloaded PDA showed a diameter of 178 ± 5 nm, while the PDA loaded with curcumin had a diameter of 290 ± 7 nm. The frequency distribution shows the number of nanoparticles in each range, both PDA and Curc-PDA, with a clear area of overlap between these structures (Fig. 2C). DLS analysis showed that PDA nanoparticles exhibited sizes ranging from 100 to 185 nm, with an average diameter of 144 nm. In contrast, the Curc-PDA displayed larger sizes, ranging from 150 to 300 nm, with an average diameter of 223 nm (Fig. 2D). Both samples showed polydispersity, evidenced by values between 0.26 and 0.37 respectively.

To determine the amount of curcumin in the PDA, we measured curcumin by UHPLC-MS, releasing it from the PDA with the addition of methanol (Fig. 2E). This compound was evaluated at 369 mass–charge-ion (m/z = 369) and showed a peak at a retention time of 17.5 ± 0.5 min. Compared with the standard of Curcumin in methanol at the same concentration, we found the concentration of curcumin in the PDA was 0.65 ± 0.04 mg/ml (Fig. 2, table), from the curcumin standard and the unloaded PDA control (Additional file 1: Table S1). Given that the concentration of loaded and unloaded PDA was 1 mg/ml, the results show that for each milligram of loaded nanoparticle, we have 0.65 mg of curcumin, i.e., 65% corresponds to curcumin, while 35% corresponds to PDA.

### Release dynamics of Curc-PDA in the culture medium

**Fig. 3:**
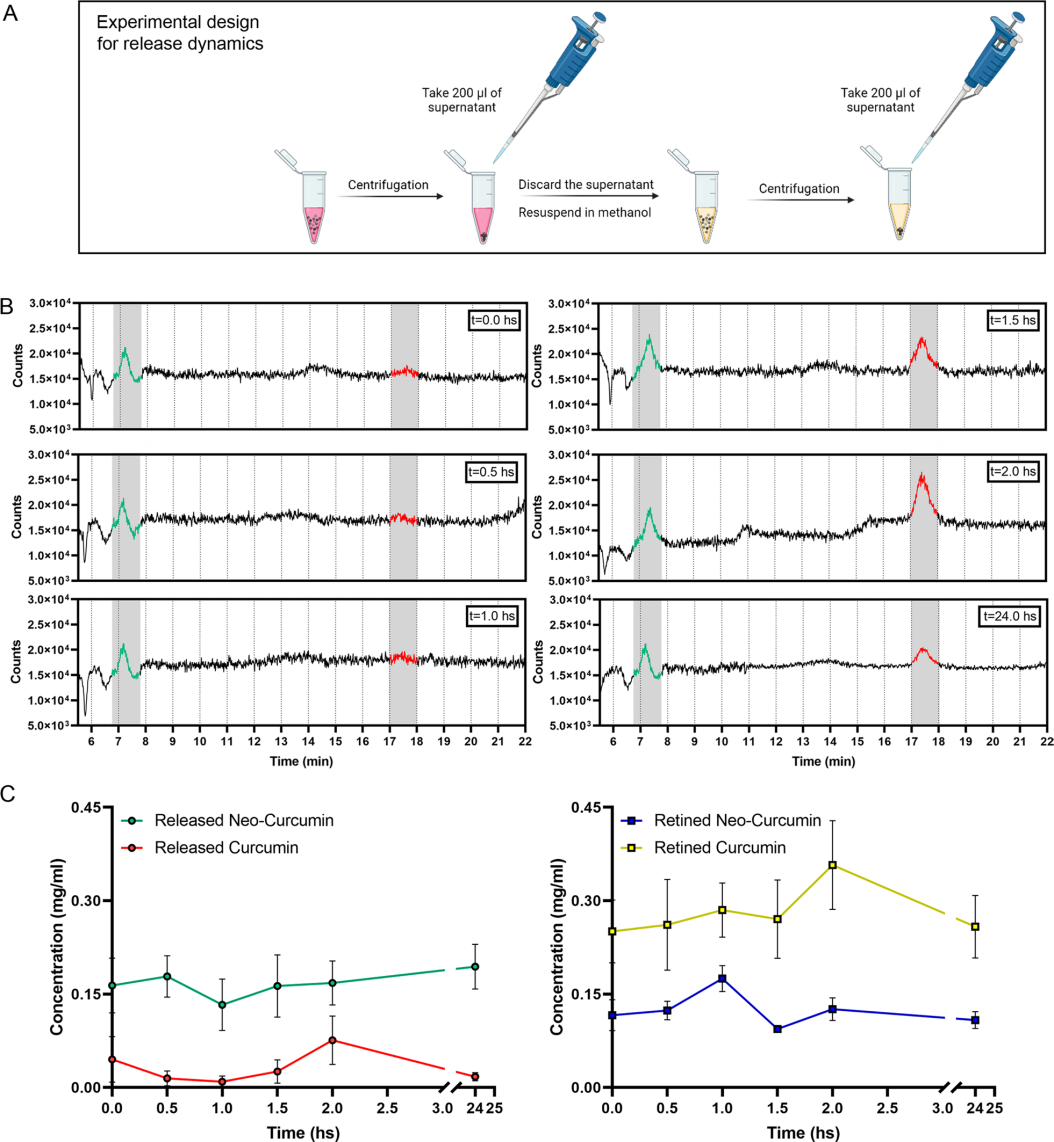
Release dynamics of Curc-PDA in the culture medium. Curcumin-loaded nanoparticles were placed in a culture medium, and the presence of curcumin released to the medium and retained in PDA was evaluated after 0.5, 1.0, 1.5, 2.0, and 24 h. **A** Experimental design created with BioRender.com. **B** Spectra obtained by UHPLC-MS, m/z = 369, for the released curcumin. Red indicates the characteristic peak of curcumin. Green means the presence of a species with the same m/z, detected at all times and in the ferulic acid standard (the latter in Additional file 1: Figure S4), which we call Neo-Curcumin. **C** Variation of the released and retained concentration and neo-curcumin concentration over time. Each point indicates the mean ± SEM

The release dynamics of curcumin were evaluated at different times: 0.5, 1.0, 1.5, 2.0, and 24 h, assessing at each time, the presence of curcumin in the culture medium by UHPLC-MS (Fig. 3).

For all times, the release diagrams show the presence of the characteristic peak of curcumin (Fig. 3A in red) at the reported retention time, but also show the presence of a new compound, with the same m/z ratio, but with a shorter retention time, 7.3 ± 0.5 min (Fig. 3A in green), which we will call neo-curcumin. Additionally, the concentrations of curcumin retained in the PDA at different times were measured by UHPLC-MS (Additional file 1: Fig. S1). The concentrations obtained from the areas under the curve are visualized in Fig. 3C. The released curcumin shows a peak of maximum concentration at 2 h and after 24 h in the medium, it is almost imperceptible. In contrast, neo-curcumin shows a concentration without variations over time, with an average of 17.5 ± 0.4 mg/ml (Fig. 3B left). In the case of retained curcumin, we found that both curcumin and neo-curcumin presented higher concentrations after 24 h (Fig. 3B right).

### Determination of the PDA concentration without-biological detectable effect in cell cultures

**Fig. 4:**
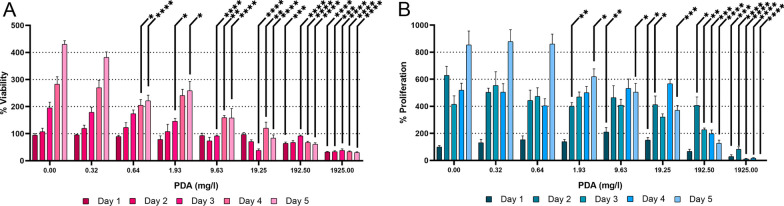
Determination of the no-effect PDA concentration in cell cultures. Before PDA loading, the maximum no-effect PDA concentration on endoneurial fibroblast cultures was determined. During the 5-day treatment, the cultures were tested every day for **A** Viability (by MTT assay) and B. Proliferation (by CyQUANT assay). The graphs show the percentage of the untreated control on day 0 (before the experiment started). Median ± SIR is plotted

To determine the maximum non-toxic PDA concentration for the cultures, we evaluated two cellular parameters: viability and proliferation (Fig. 4). The cultures were treated with different concentrations of unloaded PDA, ranging from 0.32 mg/l to 1925 mg/l. Based on the previous result of curcumin loading on PDA, these unloaded PDA concentrations allow loading from 0.1 μM to 600 μM curcumin. For each concentration tested, 5 replicates of the culture were generated, to analyze the changes of these cell parameters daily. Those wells that did not receive treatment on that day had their medium changed to the corresponding PDA concentration. The viability was measured with an MTT assay (Fig. 4A). Of the concentrations tested, only 0.32 mg/l was the concentration that showed no difference compared to the control, during the five days of treatment. The rest of the concentrations showed differences on the fifth day of treatment. The proliferation was measured with the CyQUANT assay (Fig. 4B). With this assay, we found three concentrations that did not differ from the control, 0.32, 0.64, and 1.93 mg/ml. Since 0.32 was the only concentration that coincided in both parameters, with no toxic effects on the cultures, we decided to continue our evaluation using this concentration of PDA.

### Effects on viability and proliferation of endoneurial fibroblast cultures treated with Curc-PDA.

**Fig. 5:**
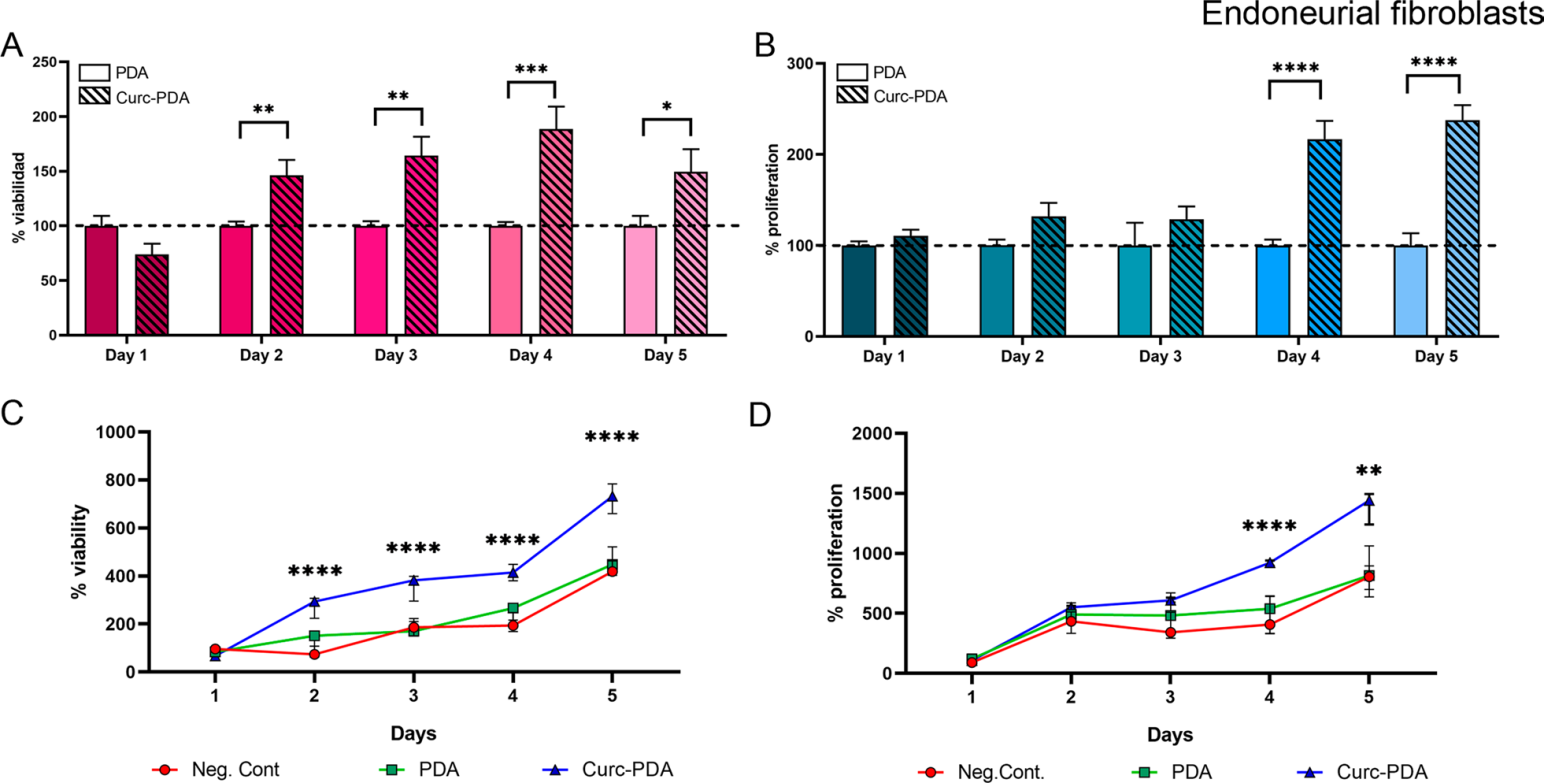
Effects on viability and proliferation of endoneurial fibroblast cultures treated with Curc-PDA. Cultures of endoneurial fibroblasts were treated with PDA (0.32 mg/l) and Curc-PDA (0.05 µM curcumin) for 5 days, assessing daily: **A** Viability (by MTT assay) and **B** Proliferation (by CyQUANT assay). The graphs show the percentage of PDA-treated cultures on the same day. **C** Cumulative viability. **D** Cumulative proliferation. Values reported for the first day of treatment. **D**. Median ± SIR is plotted

Once the working concentration of PDA was defined as non-toxic to our cultures, we evaluated the effect of curcumin treatment in PDA on the viability and proliferation of the cultures. To be below the established PDA limit, we decided to evaluate the effects of 0.05 μM curcumin (Fig. 5).

In the case of viability, from the second day of treatment, we observed an increase in the number of cells when cultures were treated with Curc-PDA (*p* = 0.002), which was maintained on the following days of treatment (day 3: *p* = 0.002; day 4: *p* = 0.001; day 5: *p* = 0.04) (Fig. 5A). If we also evaluated the percentage of accumulated growth, taking as a reference the beginning of the treatment, we find that from the second day onwards all the percentages obtained in cultures treated with Curc-PDA show significant differences with both the control without treatment and the control with unloaded PDA (*p* < 0.0001) (Fig. 5C). Comparison of the values obtained with Curc-PDA indicates a significant increase between the first and second day of treatment (*p* = 0.03), while between the second, third, fourth, and fifth day, there is no significant difference (Additional file 1: Fig. S2A).

The values obtained for proliferation showed an increase in the values with Curc-PDA during the fourth and fifth day of treatment, to the control with unloaded PDA (*p* < 0.0001) (Fig. 5B). In this sense, the evaluation of cumulative proliferation during the 5 days of treatment also shows an increase in the Curc-PDA treated cultures compared to the negative control and the unloaded PDA control on both the fourth and fifth day (day 4: *p* < 0.0001; day 5: *p* = 0.004) (Fig. 5D). These results also concur with the comparison of percentages during the whole treatment with Curc-PDA, where an increase of this parameter is visualized between the third and fourth day (*p* = 0.0002), a difference that is maintained until the fifth day (Additional file 1: Fig. S2B).

### Effects on viability and proliferation of Schwann cell cultures treated with Curc-PDA

**Fig. 6:**
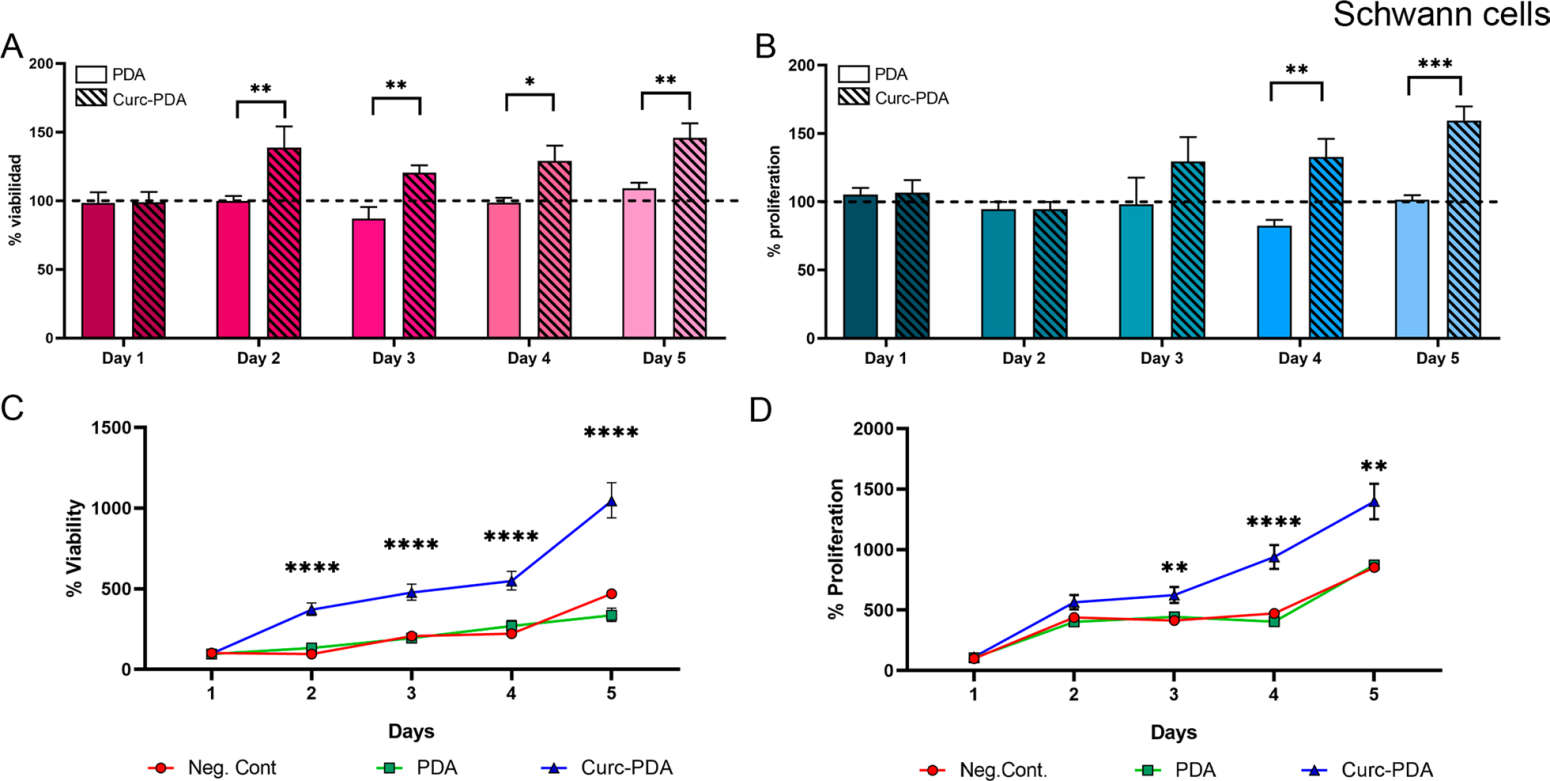
Effects on viability and proliferation of endoneurial fibroblast cultures treated with Curc-PDA. Cultures of endoneurial fibroblasts were treated with PDA (0.32 mg/l) and Curc-PDA (0.05 µM curcumin) for 5 days, assessing daily: **A** Viability (by MTT assay) and B. Proliferation (by CyQUANT assay). The graphs show the percentage of PDA-treated cultures on the same day. **C** Cumulative viability. **D** Cumulative proliferation. Values reported for the first day of treatment D. Median ± SIR is plotted

The effects of viability and proliferation on Schwann cell culture after treatment with Curc-PDA were evaluated, using the same curcumin concentration that endoneurial fibroblast (Fig. 6).

Both viability and proliferation showed the same pattern for SCs as for EFBs: compared to Curc-PDA vs PDA treatments, viability increased and then remained constant after two-day (day 2: *p* = 0.002; day 3: *p* = 0.006; day 4: *p* = 0.03; day 5: *p* = 0.009) (Fig. 6A); whereas proliferation increased after four-day treatment and maintained that difference in the last day of treatment (*p* < 0.0001) (Fig. 6B).

About the percentage of accumulated growth, we find that viability from the second day onwards all the percentages obtained in cultures treated with Curc-PDA show significant differences with both the control without treatment and the control with unloaded PDA (day 3: *p* = 0.002; day 4: *p* < 0.0001; day 5: *p* = 0.0025) (Fig. 6C).

## Discussion

In the present work, we have characterized polydopamine nanoparticles (PDA), determining their structure and their curcumin-loaded profile (Curc-PDA). In this process, we completely eliminated the use of any organic solvent to dissolve curcumin, which we consider makes a huge difference in our biological model of SCs and EFBs cell culture. In fact, we have already reported the sensitivity of SCs to the toxic action of ethanol and DMSO as a vehicle for curcumin [30].

In general, the use of curcumin is always accompanied by a solubilization stage, whatever the vehicle strategy [21, 27, 28, 52] and this introduces an extra variable in the analysis of the results obtained. This is why building nanoparticles with curcumin, without the use of organic solvents, and knowing the maximum concentration of PDA use without toxic effect on crops (0.32 mg/l), allows us to evaluate the effects and attribute them completely to the use of curcumin (0.05 μM), loaded in that harmless concentration of PDA. The approach shown in this work represents a novelty in the procedure of its delivery, where one of its main virtues is based on the elimination of the undesirable effect of the carrier used. Additionally, taking into account the pH dependence that curcumin release has shown in different contexts [49–52], we understand that the incorporation of the dialysis step after the curcumin-loaded nanoparticles has resulted in a significant improvement in the quality of the nanoparticles obtained and in their innocuousness as a carrier at the biological level.

The unloaded PDA nanoparticles were found to be smaller in size than the curcumin-loaded nanoparticles, as determined by TEM, SEM, and DLS. TEM measurements corroborated the trend observed in the DLS results (Fig. 2C and D). However, it should be noted that the particle sizes determined by TEM are larger than those determined by DLS. The DLS technique assesses the size of nanoparticles in the hydrated state, which results in a hydrodynamic diameter typically larger than the particle diameter determined by TEM [57]. The difference in our results can be attributed to the agglomeration of nanoparticles during sample preparation for TEM analysis. Incorporating a sonication step before mounting the nanoparticles on the grids could be an alternative to obtain a higher dispersion of the nanoparticles. Nevertheless, TEM and DLS showed equivalent values in their measurements.

After characterization, we evaluated the release of curcumin from PDA in a culture medium, at different times for 24 h. This allows us to identify that curcumin, detected by UHPLC-MS at a retention time of 17.5 min in the ion m/z = 369, has a peak of maximum released at 2 hs, but then its concentration decays, being very low at 24 hs. In parallel, the concentration of curcumin retained in the PDA was also analyzed by UHPLC-MS (Additional file 1: Fig. S1 in yellow). At all analyzed times, the retained curcumin concentration was higher than the released curcumin, from PDA nanoparticles. Also, we detected another peak, at a retention time of 7.3 min. This peak was detected in both the release (Fig. 3B in green) and retention analyses (Additional file 1: Fig. S1 in blue), with concentrations in both cases in the region of 0.15 mg/ml (Fig. 3C in green and blue). This compound has the same mass charge as curcumin, m/z = 369, with the retention time on the column being different. This peak, which we call neo-curcumin, is not present in the chromatographic profile control only with medium culture, nor with unloaded PDA (1 mg/ml) (Additional file 1: Fig. S3). Since neo-curcumin has the same mass, it is possibly a rearrangement or conformational change of the molecule, without chemical changes, such as a curcumin rotamer or tautomer. Chatterjee et al., 2022, recently reported the existence of rotamers in curcumin [58]. Based on that, we can hypothesize that in our loading conditions, the neo-curcumin released at 7.3 min corresponds to a rotamer of lower hydrophobicity than the “cannonic” curcumin, released at 17.5 min. To understand where this neo-curcumin peak was coming from, we decided to evaluate the standard of the major degradation product of curcumin, ferulic acid [59, 60]. To do so, we studied its ion m/z = 195 and the corresponding ion-curcumin (Additional file 1: Fig. S4). The obtained results for the standard ferulic acid (1 mg/ml in methanol) indicate the presence of a peak at the m/z 369 ion, similar to that found in neo-curcumin.

Neo-curcumin and curcumin represent the highest percentage of compounds obtained from curcumin initially loaded on PDA nanoparticles (88.17 ± 1.74%). However, between the two, neo-curcumin represents the largest component released (about 85% of the total released), in a sustained manner over time. Concerning the biological impact of curcumin delivered, we hypothesize that the effects on viability and proliferation observed in SCs and EFBs may be mainly due to these forms of curcumin released from the Curc-PDA nanoparticles. We do not rule out the possibility of characterizing the presence of other curcumin derivatives, which, given their low concentration, represent a challenge.

Exposure of cell culture to 0.05 μM curcumin in PDA for 5 days allowed us to observe changes in viability and proliferation (Fig. 5). These changes appeared with a lag time but, when they increased, they were maintained over time: while the increase in viability occurred on the second day of treatment, proliferation increased on the fourth day of treatment for SCs and EFBs (Additional file 1: Fig. S2). However, both parameters did not increase after the initial ones and remained constant until the end of treatment. This result shows that proliferation and also viability respond to the impact of curcumin action in a limited and stable manner, in long-term treatments.

As the viability assay used is the MTT, the results allow us to consider a scenario in which curcumin initially has an impact on the metabolic activity level, including mitochondrial activity or its modulation, increasing the viability. Subsequently, perhaps the direct action of curcumin and/or the accumulative effect of the metabolic and mitochondrial implications, changes in proliferation are observed at the end of the treatment in both cellular types. Moreover, in SCs, we have already reported an increase in ribosomes due to the action of curcumin [30]. In this regard, mTOR is one of the proteins that could connect mitochondrial activity with protein synthesis processes, on which biosynthesis and cell proliferation depend [61]. For this reason, our future work aims to determine the expression of this multiprotein complex to reveal through which mechanisms these processes are connected.

Regarding the dose–response phenomena, called hormesis, our results can be classified within the beneficial effects observable at low concentrations of the compound, in this case, curcumin, applied for prolonged periods. This is because there are reports of the use of working concentrations, similar to ours, related to different beneficial effects in cultures: decreases in reactive oxygen species (ROS) [33], increase in members of the chaperone response pathways, autophagy and mTOR [12, 30], and cell regeneration increase [38, 62]. In contrast, doses in cultures from 40 µm onwards show clear lethal effects, applying curcumin as a potent antitumor [8, 20].

## Conclusion

Given this broad spectrum of curcumin’s action, our main contribution lies in the characterization of a harmless curcumin’s vehicle, which guarantees not only that the observed effects can be related to the curcumin and their derivatives supply, but also that they allow a sustained and predictable release over time. Also, our findings contribute to the characterization of nanoparticles as a tool of particular value for in vitro studies focused on the dose/effect relationship over prolonged periods, for drug research and therapeutic purposes. In our current approach, it has allowed us to accurately assess the hormetic effect of curcumin.

### Supplementary Information

**Additional file 1: Table S1.** Curcumin concentration in controls. Standard curcumin and unloaded PDA, both at 1mg/ml in methanol, were evaluated to obtain the curcumin concentration from the area of integration. **Figure S1.** Curcumin retention in Curc-PDA. **Figure S2.** Change in viability and proliferation of cultures treated with Curc-PDA. **Figure S3.** Controls used for UHPLC. Figure S4. Standard evaluated by UHPLC-MS.  

## Data Availability

Data available on request due to restrictions e.g., privacy or ethical. The data presented in this study are available on request from the corresponding author.
