# Supplementary material for: A low dose of curcumin-PDA nanoparticles improves viability and proliferation in endoneurial fibroblasts and Schwann cell cultures
Source: Discov Nano. 2024 May 7;19(1):81. doi: 10.1186/s11671-024-04023-7 (PMC11076434; doi:10.1186/s11671-024-04023-7)
Supplement: Supplementary file 1 — Additional file 1: Table S1. Curcumin concentration in controls. Standard curcumin and unloaded PDA, both at 1mg/ml in methanol, were evaluated to obtain the curcumin concentration from the area of integration. Figure S1. Curcumin retention in Curc-PDA. Figure S2. Change in viability and proliferation of cultures treated with Curc-PDA. Figure S3. Controls used for UHPLC. Figure S4. Standard evaluated by UHPLC-MS. [file 11671_2024_4023_MOESM1_ESM.pdf]

Supplementary Information

Low hormetic dose of curcumin-PDA nanoparticles improves viability and proliferation in cell culture

Lucia Vázquez Alberdi[1,2] (L.V.A), Marcela Martínez-Busi[3] (M.M.B), Eloisa Arrarte[4] (E.A), Carolina Echeverry[5] (C.E.), Miguel Calero[6] (M.C) and Alejandra Kun[1,7*] (A.K.).*

Table S1. Curcumin concentration in controls. Standard curcumin and unloaded PDA, both at 1mg/ml in methanol, were evaluated to obtain the curcumin concentration from the area of integration.

Controls	Area	Concentration (mg/ml)
**PDA**	450 ± 94	0.002 ±0,001
**Curcumin**	213,599 ± 18,597	1.03 ± 0.03

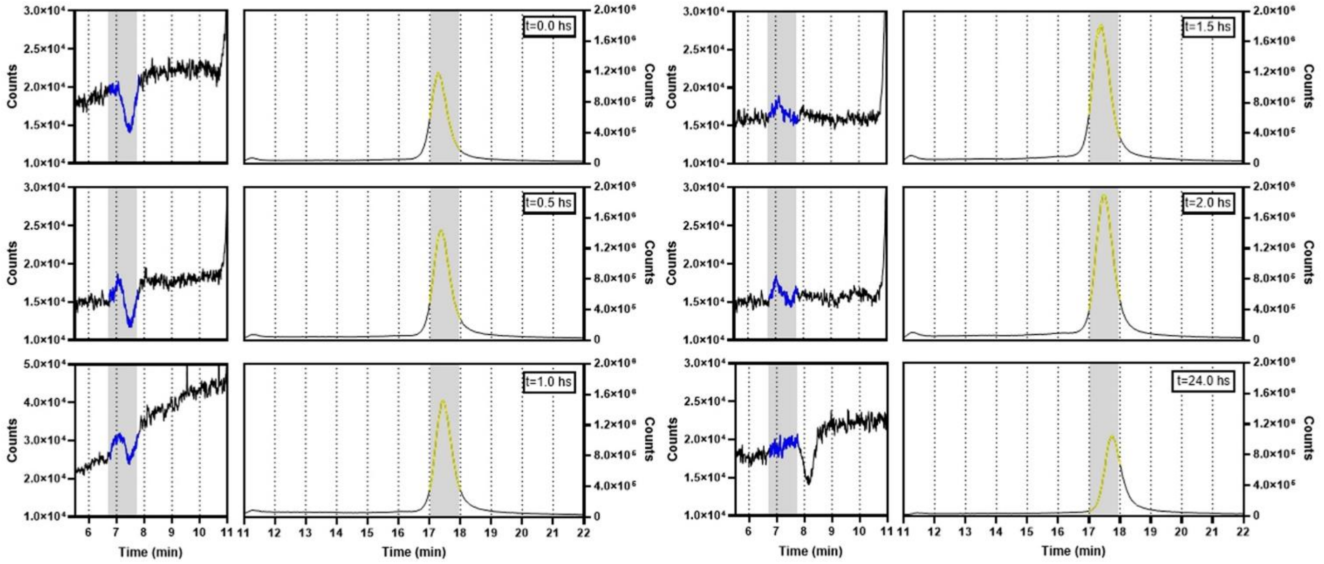

Figure S1. Curcumin retention in Curc-PDA. Curcumin-loaded NPs were placed in a culture medium, and the presence of curcumin retained in PDA was evaluated after 0.5, 1.0, 1.5, 2.0, and 24 hs. A. Spectra obtained by UHPLC-MS, m/z=369, for retained curcumin. Yellow indicates the characteristic peak of curcumin. In blue indicates the area where Neo-curcumin is found.

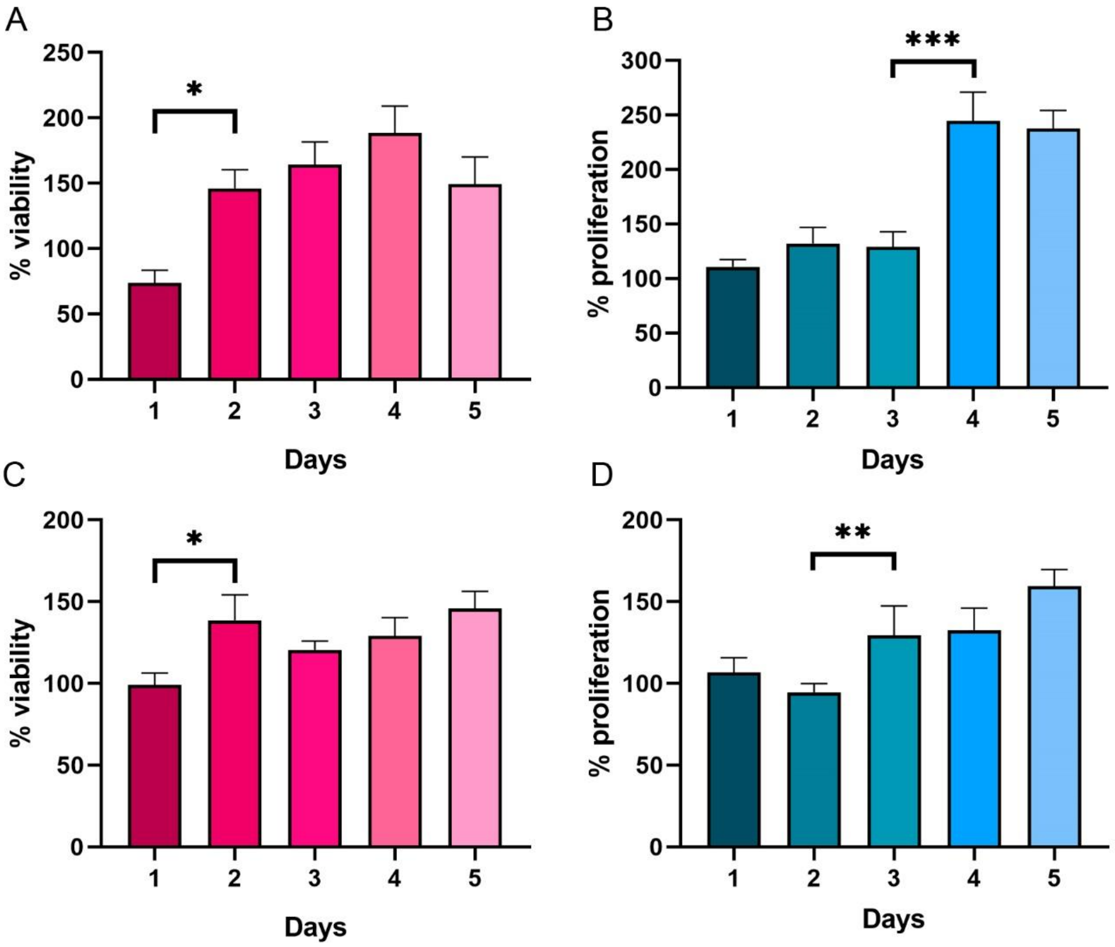

Figure S2. Change in viability and proliferation of cultures treated with Curc-PDA. For cultured treated with Curc-PDA (0.05µM curcumin) for 5 days, we assessed during which day the change in viability (by MTT assay) and proliferation (by CyQUANT assay). A. Endoneurial fibroblast cultures viability. B. Endoneurial fibroblast cultures proliferation. C. Schwann cell culture viability. D. Schwann cell culture proliferation. The median ± SEM is represented.

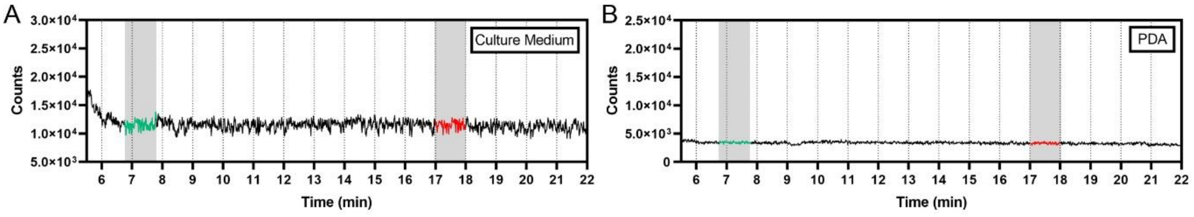

Figure S3. Controls used for UHPLC. We performed controls with A. culture medium and B. unloaded PDA (1mg/ml) at ion m/z=369 to rule out that the peaks resulting from our analysis could be due to these compounds.

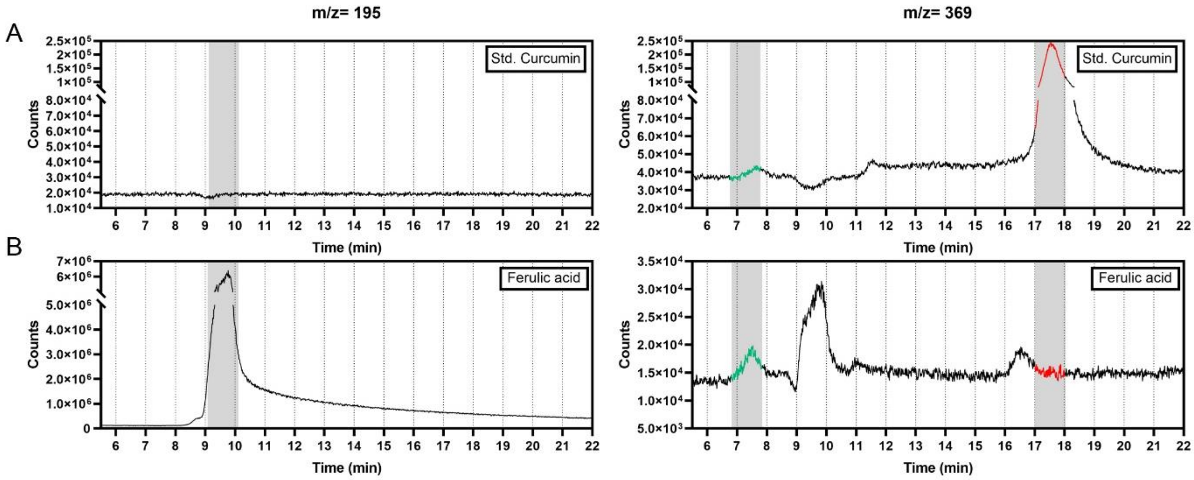

Figure S4. Standard evaluated by UHPLC-MS. The standard of A. curcumin and B. ferulic acid were evaluated at two ion m/z: 195 and 369. For this purpose, 1 mg/ml solutions in methanol were made. m/z=369 ion, the characteristic peak of curcumin is indicated in red. Green indicates the presence of Neo-curcumin.
